# The impact of a high-quality basic life support police-based first responder system on outcome after out-of-hospital cardiac arrest

**DOI:** 10.1371/journal.pone.0233966

**Published:** 2020-06-02

**Authors:** Mario Krammel, Elisabeth Lobmeyr, Patrick Sulzgruber, Markus Winnisch, David Weidenauer, Michael Poppe, Philip Datler, Sebastian Zeiner, Markus Keferboeck, Jakob Eichelter, Thomas Hamp, Thomas Uray, Sebastian Schnaubelt, Alexander Nuernberger

**Affiliations:** 1 Department of Anesthesiology, Intensive Care and Pain Medicine, Medical University of Vienna, Vienna, Austria; 2 PULS–Austrian Cardiac Arrest Awareness Association, Vienna, Austria; 3 Emergency Medical Service, Vienna, Austria; 4 Department of Emergency Medicine, Medical University of Vienna, Vienna, Austria; 5 Division of Cardiology, Department of Medicine II, Medical University of Vienna, Vienna, Austria; 6 Department of Trauma Surgery, Medical University of Vienna, Vienna, Austria; Erasmus Medical Center, NETHERLANDS

## Abstract

**Background:**

Laypersons’ efforts to initiate basic life support (BLS) in witnessed Out-of-Hospital Cardiac Arrest (OHCA) remain comparably low within western society. Therefore, in order to shorten no-flow times in cardiac arrest, several police-based first responder systems equipped with automated external defibrillators (Pol-AED) were established in urban areas, which subsequently allow early BLS and AED administration by police officers. However, data on the quality of BLS and AED use in such a system and its impact on patient outcome remain scarce and inconclusive.

**Methods:**

A total of 85 Pol-AED cases were randomly assigned to a gender, age and first rhythm matched non-Pol-AED control group (n = 170) in a 1:2 ratio. Data on quality of BLS were extracted via trans-thoracic impedance tracings of used AED devices.

**Results:**

Comparing Pol-AED cases and the control group, we observed a similar compression rate per minute (p = 0.677) and compression ratio (p = 0.651), mirroring an overall high quality of BLS administered by police officers. Time to the first shock was significantly shorter in Pol-AED cases (6 minutes [IQR: 2–10] vs. 12 minutes [IQR: 8–17]; p<0.001). While Pol-AED was not associated with increased sustained return of spontaneous circulation (p = 0.564), a strong and independent impact on survival until hospital discharge (adj. OR: 1.85 [95%CI: 1.06–3.23; p = 0.030]) and a borderline significance for the association with favorable neurological outcome (adj. OR: 1.58 [95%CI: 0.96–2.89; p = 0.052) were observed.

**Conclusion:**

We were able to demonstrate an early start and a high quality of BLS and AED use in Pol-AED assessed OHCA cases. Moreover, the presence of Pol-AED care was associated with better patient survival and borderline significance for favorable neurological outcome.

## Introduction

During the last decades, there have been several interventions trying to improve survival and neurological outcome after OHCA. The chain of survival mirrors a well-established and evidence-based treatment approach for OHCA patients and comprises early access of cardiac arrest victims, immediate cardiopulmonary resuscitation (CPR) and defibrillation, as well as professional advanced life support and post resuscitation care. However, basic life support (BLS) including the immediate initiation of sufficient chest compressions and early defibrillation using AEDs remains the basis for successful patient outcome. [1–3]

Unfortunately, rates of initiated BLS by laypersons who witness OHCA remain comparably low despite many educative interventions in this field. Therefore, in order to shorten time from the actual happening of cardiac arrest (CA) until the initiation of BLS, first responder capacities have been expanded involving government organization officials including fire fighters or police officers. [4, 5] Since police patrol cars equipped with AEDs are an easily dispatchable and fast acting instrument, a police-based first responder system (Pol-AED) has been established in various urban areas. [5] However, profound data on the impact of a Pol-AED system on patient outcome after OHCA remain scarce and inconclusive. Moreover, there is no data available on the quality of BLS administered by police officers. Considering this gap of evidence, we aimed at elucidating the quality of BLS and AED-use by police officers in the urban area of Vienna (Austria) and its impact on patient outcome after OHCA.

## Methods

### Study design and setting

A detailed protocol of the study design has already been described elsewhere. [6]

In short, the current study was conducted as a prospective population-based observational trial as part of the Vienna Cardiac Arrest Registry (VICAR). Patients suffering OHCA presenting with an initially shockable ECG rhythm (ventricular fibrillation or pulseless ventricular tachycardia) that received resuscitative attempts by the Emergency Medical Service (EMS) of Vienna (Austria) between August 2013 and September 2015 were included. Patients with obvious signs of death were not eligible for inclusion. To allow comparability of cases, only patients presenting with an initially shockable ECG rhythm were included within the current analysis. Additionally, EMS-witnessed OHCA cases were excluded from the present investigation.

The current study was conducted with waiver of informed consent due to minimal risk for participants. The protocol conforms to the Declaration of Helsinki and was approved by the local ethics committee of the Medial University of Vienna, Austria (No.1221/2013).

### Police first responder system in Vienna

Since 2013, a total of 104 AED devices have been positioned at 90 Viennese police stations. Additionally, a total of 105 police patrol cars have been equipped with AEDs leading to a total of 209 AED devices available to the municipal police service of Vienna. The following AED devices were in use: CRplus (Physio-Control, Redmond, WA, USA), HeartStart FRx (Philips, Amsterdam, Netherlands) and HeartStart HS1 (Philips, Amsterdam, Netherlands). Police officers are continuously instructed in basic life support (BLS) during 4 hours of training per year in accordance to recommendations of the European Resuscitation Council. [3]

In case of OHCA, each police car being situated in a radius of 3 kilometres (located via GPS) of the CA incident is dispatched to the scene (in addition to the EMS team) regardless any other factors. If police officers arrive before EMS, they initiate resuscitative attempts including AED use to bridge no-flow time until EMS arrives at the scene. Police officers are advised to perform chest-compression-only CPR without rescue breaths, based on recent data on this issue [7, 8] and the goal of achieving high compliance and motivation among the force. Through this, a high chest compression ratio and a decrease in no-flow time was sought to be achieved.

### Methods and data acquisition

Patient data and demographic characteristics were obtained by specially trained chart reviewers via run reports and EMS event documentation at the time of OHCA, and inserted in a predefined record abstraction form. Data acquisition was performed in accordance to the Utstein style criteria. [9] To evaluate quality of both basic- and advanced life support performance, data of trans-thoracic impedance measures and vital parameters (SpO2, etCO2, heart rate and blood pressure) were extracted out of respective defibrillator files and evaluated by trained personnel and physicians using CODE-STAT Reviewer software package (Physio-Control, Redmond, WA, USA) and Philips Heart Start Event Review (Philips, Amsterdam, Netherlands).

### Outcome measure

Survival until hospital discharge was set as the primary outcome, and sustained return of spontaneous circulation (sROSC) after OHCA, survival until hospital discharge, 30-day survival and favorable neurological outcome were chosen as the secondary study endpoints. To gather endpoint data, all enrolled participants were followed-up for 30 days after the index-event. Data were obtained by contacting patients or the respective treating physicians and screening of the patients’ hospital discharge letters. SROSC was defined as any return of cardiac output for a minimum duration of 20 minutes. Survival until hospital discharge was defined as survival until discharge from the admitting hospital to the patients’ home, nursing facility, other treating hospital or palliative care institution. The patients’ neurological functioning was evaluated as recommended according to the cerebral performance category (CPC) at the time of hospital discharge and validated after the 30-day follow-up period. [9] Good neurological outcome was defined as CPC 1 or 2. CPC was determined using a standardized follow-up procedure that consists of a clinical examination, thorough screening laboratory and clinical data, and a personal communication with the patient. This follow-up procedure was re-done after 30 days, and therefore validated the previous results. Moreover, the patients’ cause and date of death were assessed for all study participants and validated via the Austrian National Registry of Death (Statistics Austria, Vienna, Austria).

### Statistical analysis and randomization

Police AED cases were randomly assigned to a gender, age, witnessed CA and first-rhythm matched non-Pol-AED control group in a 1:2 ratio using a predefined electronic randomization software at the Medical University of Vienna. Discrete data are presented as counts and percentages and were analyzed using testing for linear association among intervals (Maentel–Haenszel chi-square test). Continuous variables are shown as medians and their respective interquartile range (IQR) and were compared using Kruskal–Wallis test. Binary Logistic Regression analysis were used to assess the impact of Pol-AED on outcome measures and were presented as odds ratio (OR) and the respective 95% confidence interval (CI). ORs were adjusted for potential confounders: age, gender, witnessed CA and location of CA. Statistical significance was defined by two-sided p-values of <0.05. Statistical analyses were performed using SPSS 24.0 (IBM SPSS, USA).

## Results

During the observation period, 2466 individuals suffering OHCA in Vienna were registered in the VICAR. Altogether, there were about 200 cases of police arriving at scene. Of these, a total of 85 Pol-AED cases with an initially shock-able rhythm were identified (the low number of cases may result in EMS arriving before-, simultaneously-, or shortly after the police car–in these cases EMS naturally took over defibrillation), and compared to a matched control group of 170 cases in which police did not act as first responder (those were attended to by EMS). Detailed baseline characteristics of the study population and stratified by Pol-AED and non-Pol-AED cases are illustrated in Table 1.

**Table 1:** Baseline characteristics.

	Police AED (n = 85)	No Police AED (n = 170)	p-value
Age, years (IQR)	64 (53–73)	64 (53–74)	0.982
Male gender, n (%)	74 (87.1)	148 (87.1)	0.999
Location of CA			0.097
Home, n (%)	39 (45.9)	88 (51.5)	
Public, n (%)	46 (54.1)	82 (48.5)	
Witnessed CA, n (%)	56 (65.9)	121 (73.7)	0.195
Time to FMC, minutes (IQR)	8 (6–11)	7 (5–9)	**0.027**
Time to first Shock, minutes (IQR)	6 (2–10)	12(8–17)	**<0.001**
Total Shocks, n (IQR)	2 (1–4)	3 (1–7)	0.064
Compression Rate/minute (IQR)	108 (102–116)	108 (102–117)	0.677
Compression Ratio, % (IQR)	82 (75–86)	83 (77–86)	0.651
Ventilation Rate/minute (IQR)	- (-)*	7 (5–9)	-
Cardiac Arrest Center Care, n (%)	27 (47.4)	55 (47.4)	0.996

Categorical data are presented as counts and percentages, continuous data as medians and IQRs. Categorical data are analyzed using a test for linear association (Maentel–Haenszel chi-square test), continuous data using Kruskal–Wallis test for testing within the subgroups. CA = Cardiac Arrest; CPR = Cardio Pulmonary Resuscitation; FMC = First Medical Contact; *Police officers were advised to perform chest compression only.

Summarized, the median patient age was 64 (53–74) years, including 148 (87.1%) male participants. The majority of CA cases occurred at home and were less likely to be witnessed in the Pol-AED group (Pol-AED: 65.9% vs. non-Pol-AED: 73.7%; p = 0.195).

### Quality of BLS and AED use

Comparing Pol-AED cases and the control group, we observed a similar compression rate per minute (p = 0.677) and compression ratio (p = 0.651), mirroring an overall high quality of BLS care administered by police officers. The time to first medical contact (defined as time EMS personnel arrived at the scene) was significantly longer in Pol-AED cases (Pol-AED: 8 minutes [IQR: 6–11] vs. non-Pol-AED: 7 minutes [IQR: 5–9]; p = 0.025). The time until the first defibrillation shock was significantly shorter in Pol-AED cases (Pol-AED: 6 minutes [IQR: 2–10] vs. non-Pol-AED: 12 minutes [IQR: 8–17]; p<0.001) and borderline significance for a lower total amount of shocks until ROSC was observed (p = 0.064).

### Outcome analysis

**Fig 1:**
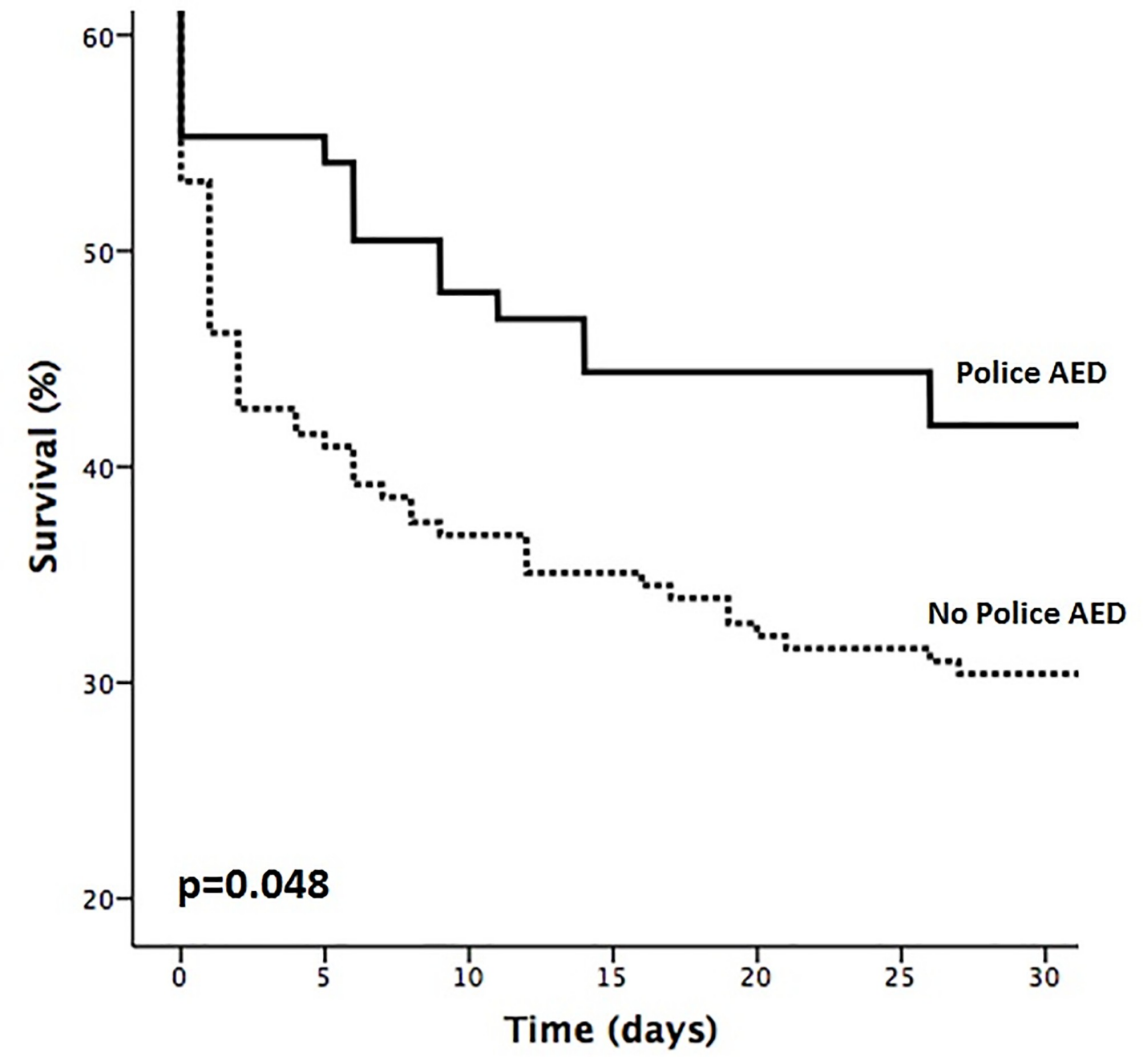
Kaplan-Meier survival plot comparing patients receiving police AED to patients without police AED using log rank test (p = 0.048). AED = automated external defibrillation.

While we observed comparable rates of sROSC in both groups (Pol-AED: 50.6% vs. non-Pol-AED: 48.2%; 0.691), a significantly higher survival until hospital discharge was observed in the Pol-AED subgroup (Pol-AED: 42.4% vs. non-Pol-AED: 29.4%; p = 0.036). Moreover, a borderline significant increase in favorable neurological outcome was observed for Pol-AED care (Pol-AED: 77.8% vs. non-Pol-AED: 74.0%; p = 0.050) (see Table 2). Although Pol-AED was not associated with sROSC with an adjusted odds ratio (OR) of 0.86 (95%CI: 0.51–1.48; p = 0.605), Pol-AED had a strong and independent impact on survival to hospital discharge (adj. OR: 1.85 [95%CI: 1.06–3.23]; p = 0.030]) and a borderline significance for the association with favorable neurological outcome (adj. OR: 1.58 [95%CI: 0.96–2.89]; p = 0.052) (see Table 3). The effect of Pol-AED on survival was confirmed using log-rank test (p = 0.048; see Fig 1).

**Table 2:** Outcome analysis.

	Total Study Population	Police AED (n = 85)	No Police AED (n = 170)	p-value
Sustained ROSC, n (%)	125 (49.0)	43 (50.6)	82 (48.2)	0.691
Survival to Discharge, n (%)	86 (33.7)	36 (42.4)	50 (29.4)	**0.036**
CPC 1 and 2, n (%)	65 (25.5)	28 (32.9)	37 (21.8)	0.054

Categorical data are presented as counts and percentages, continuous data as medians and IQRs. Categorical data are analyzed using a test for linear association (Maentel–Haenszel chi-square test) within the subgroups. ROSC = Return of Spontaneous Circulation; CPC = Cerebral Performance Category.

**Table 3:** Regression analysis.

	Sustained ROSC		Survival to Discharge		CPC 1 and 2	
	*Adj*. *OR (CI 95%)*	*p-value*	*Adj*. *OR (CI 95%)*	*p-value*	*Adj*. *OR (CI 95%)*	*p-value*
Police AED	0.86 (0.51–1.48)	0.605	1.85 (1.06–3.23)	**0.030**	1.58 (0.96–2.89)	0.052

Binary logistic regression analysis for the impact of police AED use on sustained ROSC, survival to discharge and favorable neurological outcome. The multivariate model was adjusted for: age, gender, witnessed CA and location of CA. ROSC = Return of Spontaneous Circulation; CPC = Cerebral Performance Category.

## Discussion

To the best of our knowledge, the present study represents the first in literature that demonstrated an overall high BLS and AED use quality in Pol-AED cases. Of utmost importance, we found that Pol-AED care was significantly associated with survival, and a borderline significance for favorable neurological outcome was detected. While the observed effect on survival mirrors international findings in pooled analyses [5], we present the novel results of an association of Pol-AED with a strong trend for increased neurological outcome. Mainly, we think that the police-AED program does not lead to a higher rate of ROSC (in potentially already non-savable victims) but provides help for potentially savable CA victims faster. Therefore, a quick defibrillation shock and good chest compression lead to a better outcome. Malta-Hansen et al. could already demonstrate an increase of CPC 1/2 cases after having introduced first-responder programs including law enforcement, but only presented a mixed analysis of different first responder subgroups. [10]

### Time is crucial

The concept of government officials being sent to OHCA cases as first responders is not new, as has been widely introduced and accepted as part of the chain of survival. Although the initial phase of developing such a program is challenging, time to first defibrillation was internationally shown to decrease, once the system is fully functional and operating. [5] Also in the urban area of Vienna, the Pol-AED program features time advantages: the average time interval from incoming emergency call until the initiation of CPR by the EMS amounts up to roughly 11 minutes [11] (comprised of the time from call until scene-arrival and the time of scene-arrival until actual care-giving; the latter may be lengthened by e.g. climbing staircases). This timeframe of no-flow was almost halved in Pol-AED cases in the current analysis, based on an early high-quality chest compression and AED use, and therefore leaving the patient with a higher chance of survival and favorable outcome. [12] Interestingly, the Viennese Pol-AED system achieves the first shock equally fast or even faster than when put in international comparison. [5, 13–15]

### High quality CPR as the key

Remarkably, we found no difference in chest compression rate or compression ratio between professional EMS and law enforcement personnel in our study group. Supported by recent data [7, 8], focusing on chest-compression-only CPR can probably achieve both, reduced training and practice time necessary in order to produce sufficient results, and reducing low-flow or no-flow times since police officers are not healthcare professionals familiar with rescue ventilation. Law enforcement personnel might also depict a more fitness-affine population group than average, leading to potentially better CPR results in terms of quality. [16]

### Police fills in the gaps

The modern chain of survival heavily relies on bystanding laymen to initiate BLS efforts and provide AED-defibrillation. [3] However, there are hints of an overall poor knowledge and awareness concerning these key features among the layperson population, and an additional lack of AEDs in certain regions. [17, 18] Moreover, with expected bystander-rates in OHCA still being low [11], the more important additional systems like Pol-AED seem in order to step in. Since dispatched AEDs in general is a feasible concept [19], police cars widely spread over an urban area can fill in gaps of knowledge and lack of AEDs, and therefore once more be of service to citizens. As a future perspective, an even wider distribution of the Pol-AED program is contemplated and can also be expanded including other government organizations.

### Limitations

The fact that this analysis was performed as a single area study represents the major limitation of this manuscript. Therefore, a potential problem in the generalization of results applies and only mirrors quality of the local Pol-AED system. Moreover, data lack up-to-date bystander rates, clinical characteristics and details concerning post-ROSC care. However, considering recent data from Vienna that illustrated similar quality of care in admitting hospitals [20], and profound adjustment within the multivariate model, we might have been able to minimize this potential bias.

## Conclusion

Within the present investigation we were able to demonstrate an early initiation and high quality of BLS and AED use in Pol-AED assessed OHCA cases. Moreover, the presence of Pol-AED care was associated with increased patient survival and borderline significance for favorable neurological outcome after OHCA.

## References

[1] Kragholm K, Wissenberg M, Mortensen RN, Hansen SM, Malta Hansen C, Thorsteinsson K, et al Bystander Efforts and 1-Year Outcomes in Out-of-Hospital Cardiac Arrest. N Engl J Med. 2017;376:1737–47. 10.1056/NEJMoa1601891 28467879

[2] Soar J, Nolan JP, Böttiger BW, Perkins GD, Lott C, Carli P, et al European Resuscitation Council Guidelines for Resuscitation 2015: Section 3. Adult advanced life support. Resuscitation. 2015;95:100–47. 10.1016/j.resuscitation.2015.07.016 26477701

[3] Perkins GD, Handley AJ, Koster RW, Castrén M, Smyth MA, Olasveengen T, et al European Resuscitation Council Guidelines for Resuscitation 2015: Section 2. Adult basic life support and automated external defibrillation. Resuscitation. 2015;95:81–99. 10.1016/j.resuscitation.2015.07.015 26477420

[4] Saner H, Morger C, Eser P, Planta M von. Dual dispatch early defibrillation in out-of-hospital cardiac arrest in a mixed urban-rural population. Resuscitation. 2013;84:1197–202. 10.1016/j.resuscitation.2013.02.023 23518012

[5] Husain S, Eisenberg M. Police AED programs: a systematic review and meta-analysis. Resuscitation. 2013;84:1184–91. 10.1016/j.resuscitation.2013.03.040 23643893

[6] Hubner P, Lobmeyr E, Wallmüller C, Poppe M, Datler P, Keferböck M, et al Improvements in the quality of advanced life support and patient outcome after implementation of a standardized real-life post-resuscitation feedback system. Resuscitation. 2017;120:38–44. 10.1016/j.resuscitation.2017.08.235 28864072

[7] Zhan L, Yang LJ, Huang Y, He Q, Liu GJ. Continuous chest compression versus interrupted chest compression for cardiopulmonary resuscitation of non-asphyxial out-of-hospital cardiac arrest. Cochrane Database Syst Rev. 2017;3:CD010134 10.1002/14651858.CD010134.pub2 28349529 PMC6464160

[8] Perkins GD, Olasveengen TM, Maconochie I, Soar J, Wyllie J, Greif R, et al European Resuscitation Council Guidelines for Resuscitation: 2017 update. Resuscitation. 2018;123:43–50. 10.1016/j.resuscitation.2017.12.007 29233740

[9] Perkins GD, Jacobs IG, Nadkarni VM, Berg RA, Bhanji F, Biarent D, et al Cardiac Arrest and Cardiopulmonary Resuscitation Outcome Reports: Update of the Utstein Resuscitation Registry Templates for Out-of-Hospital Cardiac Arrest: A Statement for Healthcare Professionals From a Task Force of the International Liaison Committee on Resuscitation (American Heart Association, European Resuscitation Council, Australian and New Zealand Council on Resuscitation, Heart and Stroke Foundation of Canada, InterAmerican Heart Foundation, Resuscitation Council of Southern Africa, Resuscitation Council of Asia); and the American Heart Association Emergency Cardiovascular Care Committee and the Council on Cardiopulmonary, Critical Care, Perioperative and Resuscitation. Resuscitation. 2015;96:328–40. 10.1016/j.resuscitation.2014.11.002 25438254

[10] Malta Hansen C, Kragholm K, Pearson DA, Tyson C, Monk L, Myers B, et al Association of Bystander and First-Responder Intervention With Survival After Out-of-Hospital Cardiac Arrest in North Carolina, 2010–2013. JAMA. 2015;314:255–64. 10.1001/jama.2015.7938 26197186

[11] Nürnberger A, Sterz F, Malzer R, Warenits A, Girsa M, Stöckl M, et al Out of hospital cardiac arrest in Vienna: incidence and outcome. Resuscitation. 2013;84:42–7. 10.1016/j.resuscitation.2012.07.002 22796542

[12] Vukmir RB. Survival from prehospital cardiac arrest is critically dependent upon response time. Resuscitation. 2006;69:229–34. 10.1016/j.resuscitation.2005.08.014 16500015

[13] Ross P, Nolan J, Hill E, Dawson J, Whimster F, Skinner D. The use of AEDs by police officers in the City of London. Automated external defibrillators. Resuscitation. 2001;50:141–6. 10.1016/s0300-9572(01)00343-4 11719140

[14] White RD, Asplin BR, Bugliosi TF, Hankins DG. High discharge survival rate after out-of-hospital ventricular fibrillation with rapid defibrillation by police and paramedics. Ann Emerg Med. 1996;28:480–5. 10.1016/s0196-0644(96)70109-9 8909267

[15] Stein P, Spahn GH, Müller S, Zollinger A, Baulig W, Brüesch M, et al Impact of city police layperson education and equipment with automatic external defibrillators on patient outcome after out of hospital cardiac arrest. Resuscitation. 2017;118:27–34. 10.1016/j.resuscitation.2017.06.017 28655625

[16] Lin C-C, Kuo C-W, Ng C-J, Li W-C, Weng Y-M, Chen J-C. Rescuer factors predict high-quality CPR—a manikin-based study of health care providers. Am J Emerg Med. 2016;34:20–4. 10.1016/j.ajem.2015.09.001 26431945

[17] Schnaubelt S, Krammel M, van Tulder R, Eichelter J, Gatterer C, Chwojka C, et al Public access defibrillation is insufficiently available in rural regions—When layperson efforts meet a lack of device distribution. Resuscitation. 2018;126:e4–e5. 10.1016/j.resuscitation.2018.02.028 29501397

[18] Krammel M, Schnaubelt S, Weidenauer D, Winnisch M, Steininger M, Eichelter J, et al Gender and age-specific aspects of awareness and knowledge in basic life support. PLoS ONE. 2018;13:e0198918 10.1371/journal.pone.0198918 29894491 PMC5997304

[19] Berdowski J, Blom MT, Bardai A, Tan HL, Tijssen JGP, Koster RW. Impact of onsite or dispatched automated external defibrillator use on survival after out-of-hospital cardiac arrest. Circulation. 2011;124:2225–32. 10.1161/CIRCULATIONAHA.110.015545 22007075

[20] Schober A, Sterz F, Laggner AN, Poppe M, Sulzgruber P, Lobmeyr E, et al Admission of out-of-hospital cardiac arrest victims to a high volume cardiac arrest center is linked to improved outcome. Resuscitation. 2016;106:42–8. 10.1016/j.resuscitation.2016.06.021 27368428

